# Molecular docking and network pharmacology research on the Danggui Sini Decoction’s mechanism of action for treating erectile dysfunction

**DOI:** 10.1097/MD.0000000000040529

**Published:** 2024-11-22

**Authors:** Xinyu Yan, Yiyi Zhang, Jingwen Mo, Lindong Xu, Keyu Shi, Yi Zhou

**Affiliations:** a College of Basic Medicine, Chengdu University of Traditional Chinese Medicine, Chengdu, China.

**Keywords:** Chinese herbal medicine, Danggui Sini Decoction, erectile dysfunction, molecular docking, network pharmacology

## Abstract

Utilizing network pharmacology and molecular docking, we evaluated the possible pharmacological mechanism of Danggui Sini Decoction (DGSND) for treating erectile dysfunction (ED). DGSND’s chemical components and targets were found utilizing the Traditional Chinese Medicine Systems Pharmacology Database and Analysis Platform (TCMSP). Disease-related genes associated with ED were identified through GeneCards, OMIM, TTD, DrugBank, and DisGeNET databases. These datasets intersected to identify possible DGSND targets for treating ED. We developed an interactive visual network that linked herbs, active components, diseases, and targets using Cytoscape 3.7.1. The protein–protein interactions (PPI) were analyzed using the STRING database. The DAVID database was used to conduct gene ontology (GO) and Kyoto Encyclopedia of Genes and Genomes (KEGG) pathway enrichment studies to determine the mechanism of action of the discovered genes. The pathways most strongly associated with ED were analyzed through histograms and bubble maps. From the PPI network, the 6 promising targets were selected for molecular docking with the top ranked compounds in terms of degree value. DGSND contains 7 Chinese herbal medicines, 142 main components, and 73 latent targets for treating ED. GO and KEGG analyses suggest that DGSND may have the ability to modulate oxidative stress, apoptosis, and inflammatory responses. Through the PPI network and topology analysis, 6 core genes were pinpointed. Molecular docking revealed that beta-sitosterol exhibited the lowest binding energy with BCL2, indicating a more stable structure. This study demonstrates that DGSND’s compounds stimulate NO synthesis and reduce inflammation and cell apoptosis to improve ED by acting on AKTI, ALB, IL6, TNF, TP53, and BCL2. The findings show that DGSND’s compounds These findings offer a valuable scientific foundation for further understanding the mechanism of DGSND in treating ED.

## 1. Introduction

Erectile dysfunction (ED) is the incapacity of the penis to attain or maintain an erection sufficient for satisfactory sexual intercourse and is 1 of the common sexual dysfunctions in men.^[1]^ With over 150 million affected men worldwide, a study conducted by Chinese researchers surveyed 5210 men over the age of 40 across 30 provinces and cities in China, revealing a prevalence rate of 40.56% in this age group.^[2]^ ED is a prevalent disorder affecting around 1 in 5 American men.^[3]^ The prevalence of ED increases with age globally and continues to rise each year, highlighting the significance of this health issue. Penile erection is a complex physiological phenomenon that occurs as a result of precise cooperation and coordination between nerves, blood vessels, and tissues. Any issues in this process can result in abnormal erectile function.^[4]^ The etiological classification of ED typically includes organic, psychological, or mixed causes. Among these, the most common organic causes are vascular, neurological, endocrine, surgery, trauma, and drug-related factors.^[5]^ For instance, testosterone decreases, and the quantity and receptivity of neurotransmitters and vasoactive amines are reduced, weakening vascular function.^[6]^ Currently, Phosphodiesterase-5 (PDE-5) inhibitors are the first-line medications of ED, which includes sildenafil, tadalafil, and vardenafil.^[7]^ However, the use of these drugs may lead to visual disorders, dizziness, and sensorineural hearing loss, as well as other side effects.^[8,9]^ From a clinical perspective, it can be observed that, for the majority of patients, such drugs are only effective when taken and cannot be considered a cure for ED. Consequently, they cannot be taken consistently. Given the dearth of efficacious pharmaceuticals currently available to treat ED, it is imperative to conduct further research into potential therapeutic agents for this condition.

The Danggui Sini Decoction (DGSND), originating from Zhang Zhongjing’s Treatise on Cold Damage Diseases, consists of Angelica sinensis, Cassia twig, White paeony root, Asarum, Tetrapanacis Medulla, Jujube, and Licorice, which has the function of warming the meridians, dispelling cold, nourishing blood, and dredging pulse.^[10]^ It was designed to address the issue of cold hands and feet caused by blood stasis and cold coagulation of meridians. The hands and feet are the body’s extremities. The sensation of coldness in these areas indicates an impairment in the smooth circulation of blood to the extremities, and the meridians cannot be warmed and nourished.^[11]^ Considering that the penis is also a part of the human body, situated at the end of the limb. The physiological erection of the penis is a vascular response that is regulated by nerves, normal vascular function is the basis of the physiological erection of the penis. In order to achieve an erection, the penis requires sufficient blood flow into the cavernous body.^[12]^ Consequently, any disease that may lead to abnormal blood flow in the corpus cavernosum of the penis, including atherosclerosis, artery damage, artery stenosis, pudendal artery shunt, or cardiac dysfunction, can lead to ED.^[13]^ There is a body of evidence from clinical practice indicating that DGSND may achieve a better curative effect in treating ED.^[14]^ However, the existing research evidence is derived mainly from observational studies, and the specific mechanism and critical targets of DGSND in treating ED remain unclear. The mechanism was investigated through network pharmacology and molecular docking to elucidate the mechanism of action of DGSND in treating ED.

## 2. Materials and methods

### 2.1. Screening of effective components and targets of DGSND

DGSND contains 7 herbs: Angelica sinensis, Cassia twig, Paeony, Asarum, Tongcao, liquorice, and Jujube. The Traditional Chinese Medicine Systems Pharmacology Database and Analysis Platform^[15]^ (TCMSP) was used to determine the chemical composition of the 7 Chinese traditional medicines. According to the pharmacokinetic parameters of the obtained ingredients, the conditions were limited to oral bioavailability (OB) ≥ 30% and drug-likeness (DL) ≥ 0.18, and the active ingredients of DGSND were supplemented by searching the literature and obtaining the active ingredients of DGSND. Similarly, we identified targets related to the active ingredients of herbal medicines by TCMSP, and the target was further imported into UniProt^[16]^ to obtain matching gene symbols.

### 2.2. Collection of therapeutic targets related to ED

With “Erectile dysfunction” as the search term, related targets of ED were mined from the following resources: GeneCards,^[17]^ Online Mendelian Inheritance in Man (OMIM),^[18]^ Therapeutic Target Database (TTD),^[19]^ DrugBank,^[20]^ Disease Gene Network (DisGeNET),^[21]^ merging to get ED related therapeutic targets.

### 2.3. Screening of common targets of DGSND and ED

By combining the component targets of DGSND and ED targets, The Venny 2.1^[22]^ was utilized to create an intersection diagram that showcases the common targets between components and diseases, which is the potential targets for therapeutic intervention of Danggui Sini Decoction in treating ED.

### 2.4. “TCM-Compound-Disease-Target” pharmacological network construction

According to the results of 2.1, 2.2, and 2.3, we created network and type tables and constructed a complex target interaction network of “Herb-compound-disease-target” using Cytoscape 3.7.1.^[23]^

### 2.5. Construction and topology analysis of the PPI network

The targets screened in 2.3 were entered into the STRING database^[24]^; the conditions are set as follows: the species is “homo sapiens” with a minimum, medium confidence of 0.4000. Please save the file in TSV format, import it into Cytoscape 3.7.1, and visually analyze it with the Network Analyzer module. Additionally, CytoNCA was used in Cytoscape for topology analysis. In the CytoNCA Master Score File, each score index, such as “Betweenness,” “Closeness,” and “Degree,” exceeded the median threshold. Then, the core protein was determined based on the degree value, and the protein with a higher degree value was selected as the pivotal protein.^[25]^

### 2.6. GO and KEGG pathway enrichment analysis

The disease-drug intersection target genes selected by 2.3 were entered into the Database for Annotation, Visualization and Integrated Discovery (DAVID) database.^[26]^ The species “homo sapiens” was selected and systematically analyzed in terms of Biological Process (BP), Cellular Component (CC), Molecular Function (MF), and signaling pathway. A smaller *P*-value is indicative of a stronger association with the disease. The 10 entries with smaller *P*-values were selected from the Gene Ontology (GO) analysis results, and the 25 entries with smaller *P*-values were selected from the Kyoto Encyclopedia of Genes and Genomes (KEGG) pathway analysis results and visualized through the use of Weishengxin online mapping platform.

### 2.7. Potential active ingredient-target molecular docking validation

The TCMSP was utilized to download the chemical structures of the top 3 active ingredients in terms of degree value in MOL2 format. The core proteins obtained in 2.5 were identified by searching The RSCB Protein Data Bank (PDB) database^[27]^ (http://www.rcsb.org/). Their 3D structures were then saved as pdb format files. The obtained protein was uploaded to AutoDock 1.5.7, and its water molecules were removed, hydrogenated, then saved as a pdbqt file. Then, we imported the small molecule into AutoDock 1.5.7, added all hydrogen, set it as a ligand, and exported it into the pdbqt file layout. The pdbqt files of proteins and small molecules were imported into AutoDock 1.5.7, the docking activity coordinate positions were set according to each crystal structure, and the files were exported into the GPF format. Finally, the molecular docking was performed with AutoDock, and the binding energy results were obtained and recorded. The model with the minimum binding energy was chose for visualization with PyMOL.

## 3. Results

### 3.1. Essential compounds and targets in DGSND

In this experiment, the compounds of DGSND were collected from the TCMSP website, and the filter conditions were set as OB ≥ 30% and DL ≥ 0.18. The main compounds were obtained as follows: Angelica sinensis has 2 main ingredients, Cassia twig has 7, Peony has 13, Asarum has 8, Tetrapanacis Medulla has 4, Licorice has 92, and Jujube has 29 main ingredients. (see Table S1, Supplemental Digital Content, http://links.lww.com/MD/N887, which illustrates the DGSND’s effective compounds). According to the literature, 1 active ingredient of Angelica sinensis was added,^[28]^ the duplicate value was deleted, and 142 main active ingredients were screened. Discarding ingredients for which no target can be found and searching for the related targets of the active components by TCMSP, 265 potential targets in the DGSND were obtained. (see Table S2, Supplemental Digital Content, http://links.lww.com/MD/N888, which illustrates the DGSND’s compounds and targets).

### 3.2. Consulting and screening of ED disease targets

The GeneCards, OMIM, TTD, DrugBank, and DisGeNET databases provided 396, 394, 11, 35, and 256 disease targets, respectively. After eliminating duplicate targets, we obtained 953 target genes. (see Table S3, Supplemental Digital Content, http://links.lww.com/MD/N889, which illustrates the targets of DGSND and ED).

### 3.3. Results of intersection targets

**Figure 1.**
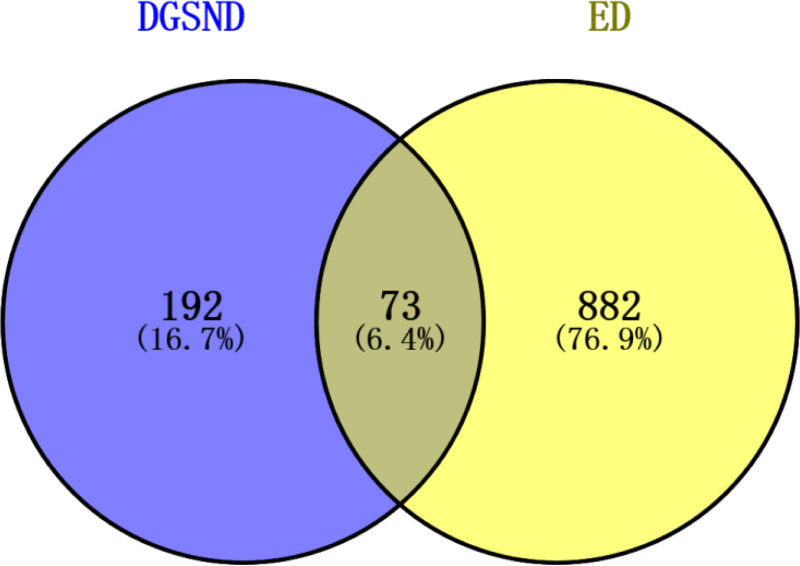
Disease-drug target intersection diagram. The blue section represents the targets for DGSND, and the yellow section represents the targets for ED. The common targets among the 2 are 73. DGSND = Danggui Sini Decoction, ED = erectile dysfunction.

Taking the intersection of DGSND’s 265 potential targets and ED’s 955 targets, we obtained 73 intersecting gene targets of DGSND and ED, and the common targets are shown in Figure 1.

### 3.4. Construction result of “TCM-Compound-Disease-Target” pharmacological network

**Figure 2.**
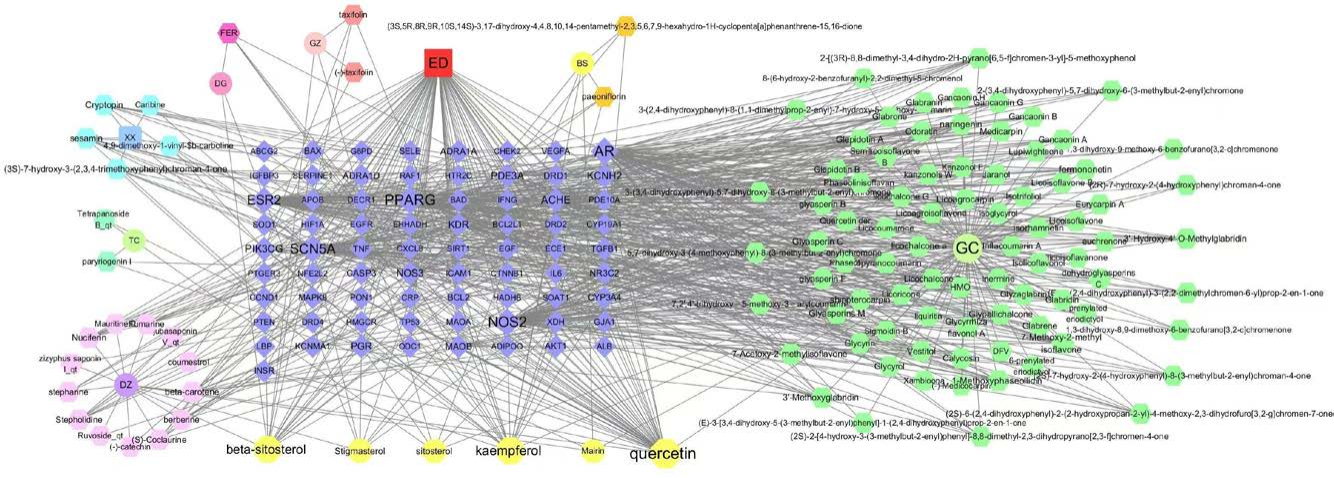
The “Herb-compound-disease-target” interaction pharmacology network. The herbs of DGSND are represented by a circle. Regular hexagons represent compounds in DGSND. Diamonds represent targets associated with ED. The edge represents the interaction between the compound and the target gene. The yellow regular octagon indicates the active ingredients shared by 2 or more Chinese medicines. BS = BaiShao. DG = DangGui. DGSND = Danggui Sini Decoction. DZ = DaZao. ED = erectile dysfunction. GC = GanCao. GZ = GuiZhi. TC = TongCao. XX = XiXin.

The data, including targets, effective ingredients, disease, and herbs, were imported into Cytoscape 3.7.1 for visual analysis. A composite pharmacological network with 194 nodes and 784 edges was generated (Fig. 2). The top 3 core active ingredients were selected according to their degree value, and detailed information is provided in Table 1. (see Table S4, Supplemental Digital Content, http://links.lww.com/MD/N890, the node table of the TCM-Compound-Disease-Target Network).

**Table 1:** Core active ingredient list.

MOL ID	Active ingredient	Degree value	Source
MOL000098	Quercetin	44	GanCao, DaZao
MOL000422	Kaempferol	19	XiXin, BaiShao, GanCao
MOL000358	Beta-sitosterol	12	DangGui, GuiZhi, BaiShao, DaZao

### 3.5. The results of the PPI network

**Figure 3.**
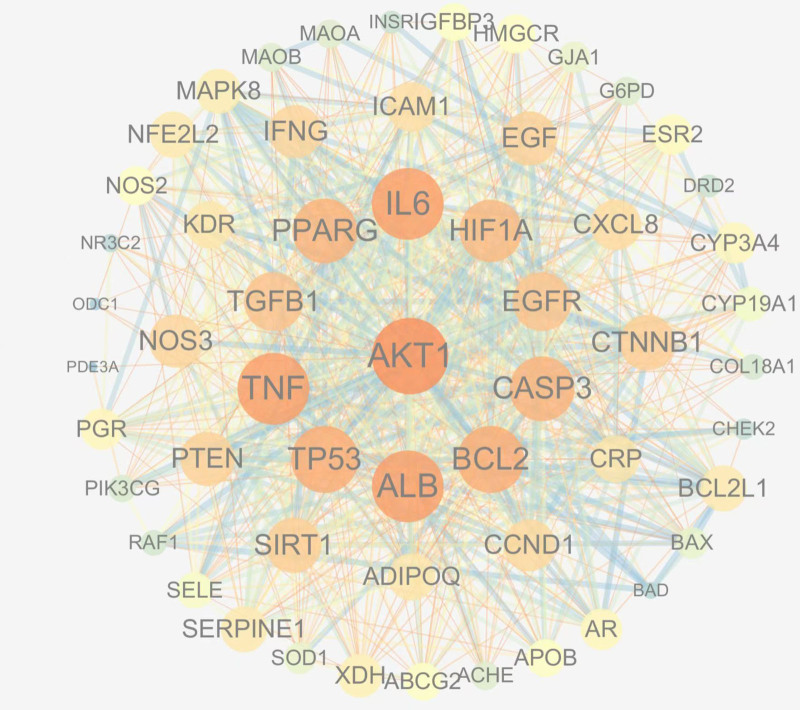
Analysis diagram illustrating the network of target-protein interactions.

The 73 genes were input into the STRING database, and the resulting data were imported into Cytoscape 3.7.1 to make the PPI network diagram (Fig. 3). The network contained 73 nodes and 811 edges, and the mean number of nodes was 22.2. Topology analysis of data was performed using the CytoNCA plug-in in Cytoscape 3.7.1, it was found that the degree values of 18 nodes AKT1, ALB, IL6, TNF, TP53, BCL2, PPARG, CASP3, HIF1A, EGFR, TGFB1, CTNNB1, PTEN, NOS3, CYP3A4, ESR2, HMGCR, and NOS2 were significantly higher than those of other targets. These proteins regulate many biological processes, including metabolism, cell survival, growth, angiogenesis, blood colloid osmotic pressure, inflammatory response, cell proliferation, and apoptosis, suggesting that they may be important targets of DGSND in treating ED. (see Table S5, Supplemental Digital Content, http://links.lww.com/MD/N891, the node table of the protein–protein interaction network analyze).

### 3.6. Enrichment analysis of GO and KEGG pathways

**Figure 4.**
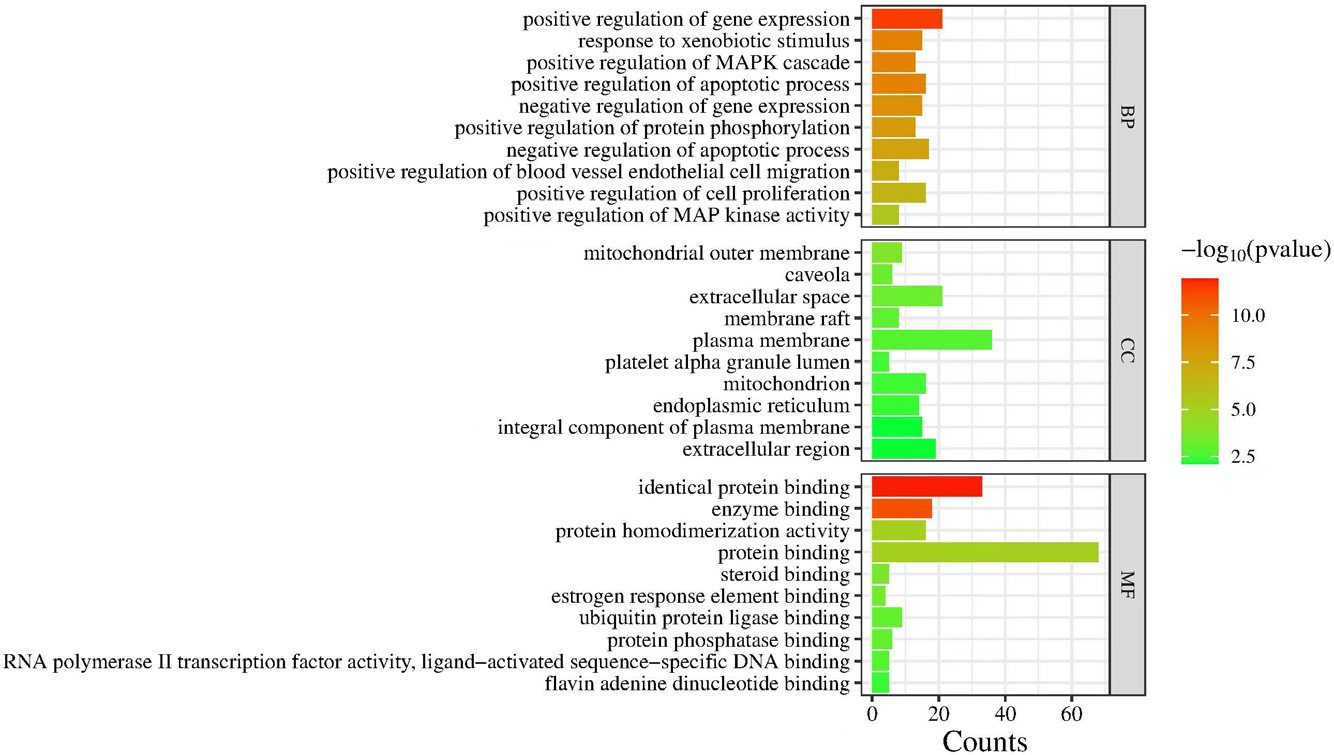
Gene ontology enrichment analysis of the target genes.

**Figure 5.**
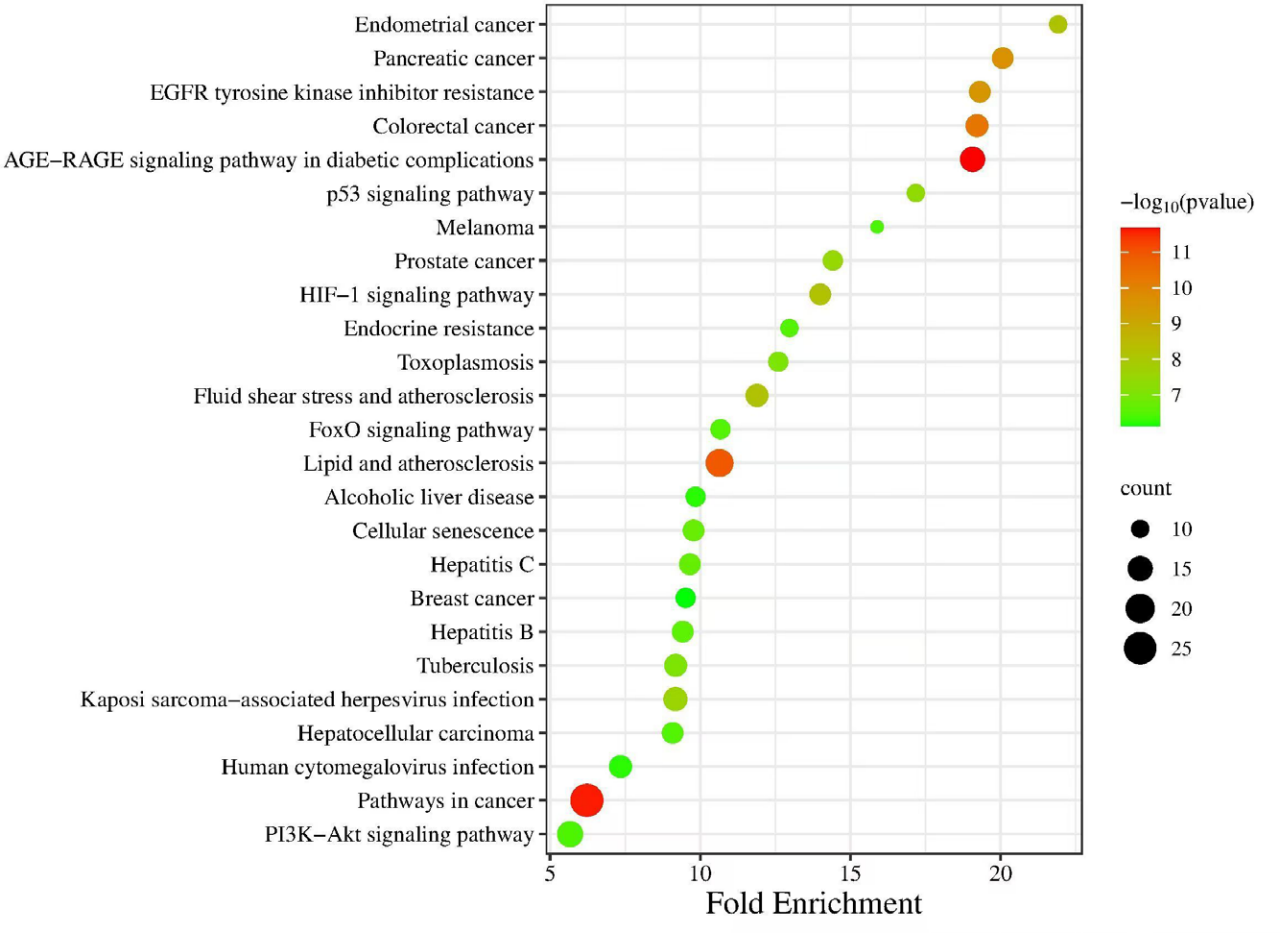
Kyoto Encyclopedia of Genes and Genomes pathway enrichment analysis.

Using the DAVID database, the common targets of DGSND and ED were analyzed for GO functional enrichment, and 217 GO items were obtained (*P* < .05; the *P*-value provided for the enrichment analysis is FDR-adjusted), including 185 BP items, 17 CC items, and 25 MF items. The most important biological functions were positive regulation of gene expression and apoptotic process. The top ten items are shown in Figure 4. There are 128 pathways screened by KEGG enrichment analysis (*P* < .05). These pathways were mainly connected with the AGE–RAGE signaling pathway in diabetic complications, pathways in cancer, lipid and atherosclerosis, colorectal cancer, fluid shear stress, atherosclerosis, prostate cancer, p53 signaling pathway, cellular senescence, FoxO signaling pathway, and PI3K–Akt signaling pathway. The first 25 terms are shown in Figure 5. (see Table S6, Supplemental Digital Content, http://links.lww.com/MD/N892, the Enrichment analysis of GO and KEGG pathways).

### 3.7. Molecular docking results

**Figure 6.**
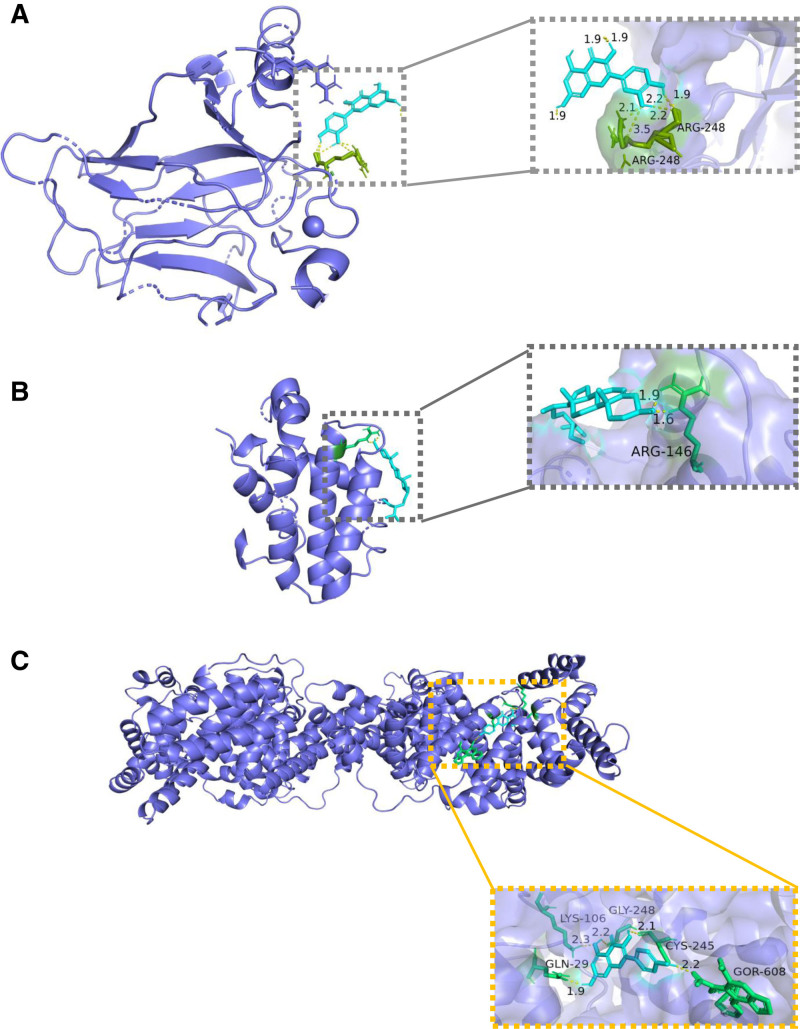
Pattern diagram of the molecular docking results. (A) quercetin-TP53, (B) kaempferol-ALB, (C) beta-sitosterol-BCL2.

The top 3 effective constituents of DGSND were quercetin, kaempferol, and β-sitosterol. These 3 important active components were chosen as the ligands. Among the 18 core targets, 6 core targets, AKT1, ALB, IL6, TNF, TP53, and BCL2, were selected as receptors for AutoDock docking based on a degree value of not <47, and the binding energy of the ligand-receptor was obtained. (see Table S7, Supplemental Digital Content, http://links.lww.com/MD/N893, the protein–protein interaction core targets). The less the binding energy value, the more stable the conformation. The binding energy values of the 3 active components of DGSND and the 6 core targets are all below −5.0 kcal/mol, indicating that they have high binding degree and stable conformation. And the binding energy score of quercetin with TP53 is lower, kaempferol with ALB is lower. β-sitosterol has a minimum binding energy with the core target BCL2. From this, it can be seen that quercetin, kaempferol, and β-sitosterol may be the crucial constituents of DGSND in treating ED. The combination of the lowest binding energy values was selected, and the visualization diagram was constructed using PyMOL 2.4. 0 (Fig. 6). The docking scores are listed in Table 2.

**Table 2:** Molecular docking results (unit: kcal/mol).

Ingredients	Targets					
	AKT1	ALB	IL6	TNF	TP53	BCL2
Quercetin	−7.94	−8.55	−5.67	−7.65	−8.59	−7.92
Kaempferol	−7.88	−8.59	−5.93	−7.99	−8.22	−8.44
Beta-sitosterol	−7.89	−7.69	−7	−9.23	−8.48	−12.23

## 4. Discussion

This study utilized network pharmacology to preliminarily investigate the mechanism of action of DGSND in the treatment of ED. A network of “TCM-Active Ingredient-Disease-Target” was constructed, which reflects the efficacy of DGSND in treating ED through multiple targets and pathways. This study found that quercetin, kaempferol, and β-sitosterol may be the key components of DGSND for ED treatment. Related literature shows that quercetin modulates the activity of enzymes and biomolecules in the penile tissue of rats with ED, such as PDE-5, angiotensin-1 converting enzyme, and malondialdehyde and nonprotein mercaptans.^[29]^ There are studies have found that kaempferol plays a major role in anti-inflammation, inhibiting peroxidation, regulating vascular tension, and anti-atherosclerosis.^[30]^ Wang et al^[31]^ found that plants containing kaempferol significantly reduced the levels of IL-6, IL-8, TNF-α, IL-1β, and IL-10 in the serum and IL-6, IL-8, and TNF-α in the prostate tissue of rats. Therefore, kaempferol has an inhibitory effect on the expression of several inflammatory factors, which in turn reduces the damage of inflammatory factors to the penile corpus cavernosum and vascular endothelial cells and improves ED. β-sitosterol has pharmacological actions such as antioxidant, anti-inflammatory, and hypoglycemic.^[32]^ Studies have shown that β-sitosterol is able to remove oxygen free radicals effectually, weaken intracellular peroxidase levels, and exert antioxidant effects. It exerts anti-inflammatory effects by inhibiting IL-6 activity in macrophages and reducing the secretion of IL-1 and TNF.^[33]^ The above studies confirmed that a variety of active ingredients in DGSND, such as quercetin, kaempferol, and β-sitosterol, could be the core compounds of DGSND for ED treatment.

GO enrichment analysis revealed that DGSND participates in the positive regulation of gene expression, apoptotic process, MAPK cascade, protein phosphorylation, blood vessel endothelial cell migration, cell proliferation, MAP kinase activity; negative regulation of gene expression, apoptotic process; and response to xenobiotic stimulus. KEGG pathway analysis proved that the active ingredients might treat ED through the AGE–RAGE signaling pathway in diabetic complications, FoxO signaling pathway, PI3K–Akt signaling pathway, etc. It plays a role in colorectal, pancreatic, prostate, and other cancer pathways. Activating the AGE–RAGE signaling pathway directly inactivates NO or indirectly reduces the production of NO,^[34]^ increases the level of vasoconstricting factors, and causes oxidative stress in cavernous tissues, resulting in increased production of vascular inflammatory factors and apoptosis of vascular endothelial cells, thus leading to the incidence of ED.^[33]^ Inhibition of the AGE–RAGE signaling pathway can inhibit the release of inflammatory mediators such as IL-1β and TNF-α, thus improving ED.^[35]^ The FoxO signaling pathway is crucial in cell cycle control, proliferation, regulation, death, and oxidative stress. Activating the PI3K–Akt–FoxO signaling pathway regulates the genes of MPG neurons to promote peripheral nerve regeneration in ED rats with cavernous nerve crush injury and restore their erectile function.^[36]^ The PI3K–Akt signaling pathway promotes metabolism, proliferation, cell survival, growth and angiogenesis. Its activation increases the NO content in smooth muscle cells of the penile cavernous, thereby improving ED.^[33,37]^

Through PPI network analysis, AKTI, ALB, IL6, TNF, TP53, and BCL2 were found to be the core targets of the interaction between DGSND and ED. AKT1 has been shown to play an essential role in regulating various processes like cell growth, division, and inhibition of apoptosis. AKT1 modulates vascular endothelial cell proliferation via PI3K-Akt. Akt1 activates the phosphorylation of eNOS and promotes the synthesis of NO.^[38]^ It plays an essential role in the stimulation of penile erection. ALB accounts for about half of the serum proteins involved in free radical scavenging and neuronal protection. It is also closely connected with systemic nutritional conditions and inflammatory responses.^[39]^ IL-6 and TNF are pro-inflammatory factors, and inflammatory factors damage endothelial cells, which results in a decrease in NO derived from the endothelial cells, leading to abnormality of the NO-cGMP pathway and further affecting penile erection function. Furthermore, irreversible endotheliocyte damage affects arterial perfusion to the penis and reduces the contractile function of the cavernous smooth muscle of the penis,^[40,41]^ thereby inducing ED.

TP53 is a tumor suppressor gene, and the p53 protein expressed by TP53 is a critical transcriptional regulator of the cell cycle, apoptosis, and DNA repair, which can protect the function of the vascular endothelium and nerves.^[42]^ p53 can cause the expression of inflammatory factors, damage endothelium-dependent vasodilation activity, and accelerate endothelial dysfunction, which is an essential pathological factor of ED.^[43]^ BCL2 is an apoptosis inhibitor. It inhibits apoptosis in a variety of cellular systems.^[44]^ PI3K/Akt and FoxO pathway are closely related with apoptosis.^[45]^ The apoptosis of nerve cells, cavernous sinus endothelial cells, and cavernous smooth muscle cells closely affect penile ED.^[46]^ Studies have shown that by increasing BCL2, apoptosis of corpus cavernosum cells can be inhibited, thereby improving ED in rats.^[47]^ Molecular docking results indicated that the main active ingredients in Angelica Sini Decoction had better binding energies with core targets, and the docking results for BCL2 and β-sitosterol were the best. Therefore, β-sitosterol holds promise as anew therapeutic agent, and BCL2 is a promising target for improving ED.

## 5. Conclusion

This study suggests that DGSND may treat ED through multiple targets such as AKTI, ALB, IL6, TNF, TP53, and BCL2. ED was treated via the AGE–RAGE signaling pathway, FoxO signaling pathway, PI3K–Akt signaling pathway, and other pathways. BCL2 may be a significant target, and β-sitosterol may be essential in treating ED. It also provides a reference for further exploration of the mechanism of action. However, in future studies, a more in-depth mechanistic discussion is needed to provide more solid basic evidence to support the treatment of ED by DGSND. In conclusion, this study preliminarily discusses the possible mechanism of DGSND in treating ED and offers some foundation for clinical utilization. Moreover, a mechanistic study based on network pharmacology will offer guidance for exploring the treatment of ED with TCM at the molecular expression level.

## Author contributions

**Data curation:** Xinyu Yan, Yiyi Zhang, Jingwen Mo.

**Formal analysis:** Xinyu Yan.

**Writing – original draft:** Xinyu Yan, Yiyi Zhang, Jingwen Mo, Lindong Xu, Keyu Shi.

**Writing – review & editing:** Xinyu Yan, Yiyi Zhang, Yi Zhou.

## Supplementary Material


