# Supplementary material for: Molecular docking and network pharmacology research on the Danggui Sini Decoction’s mechanism of action for treating erectile dysfunction
Source: Medicine (Baltimore). 2024 Nov 22;103(47):e40529. doi: 10.1097/MD.0000000000040529 (PMC11596949; doi:10.1097/MD.0000000000040529)
Supplement: Supplementary file 1 [file medi-103-e40529-s001.docx]

| **Mol ID** | **Compound** | **OB%** | **DL** | **Source** |
| --- | --- | --- | --- | --- |
| MOL000358 | beta-sitosterol | 36.91 | 0.75 | DG，GZ,BS,DZ |
| MOL000449 | Stigmasterol | 43.83 | 0.76 | DG,DZ |
| MOL000359 | sitosterol | 36.91 | 0.75 | GZ,BS,TC,GC |
| MOL000492 | (+)-catechin | 54.83 | 0.24 | GZ,BS,DZ |
| MOL000422 | kaempferol | 41.88 | 0.24 | XX,BS,GC |
| MOL000211 | Mairin | 55.38 | 0.78 | BS,GC,DZ |
| MOL000098 | quercetin | 46.43 | 0.28 | DZ,GC |
| MOL000360 | Ferulic acid | 39.56 | 0.06 | DG |
| MOL001736 | (-)-taxifolin | 60.51 | 0.27 | GZ |
| MOL000073 | ent-Epicatechin | 48.96 | 0.24 | GZ |
| MOL004576 | taxifolin | 57.84 | 0.27 | GZ |
| MOL011169 | Peroxyergosterol | 44.39 | 0.82 | GZ |
| MOL012140 | 4,9-dimethoxy-1-vinyl-$b-carboline | 65.3 | 0.19 | XX |
| MOL012141 | Caribine | 37.06 | 0.83 | XX |
| MOL001460 | Cryptopin | 78.74 | 0.72 | XX |
| MOL001558 | sesamin | 56.55 | 0.83 | XX |
| MOL002501 | [(1S)-3-[(E)-but-2-enyl]-2-methyl-4-oxo-1-cyclopent-2-enyl] (1R,3R)-3-[(E)-3-methoxy-2-methyl-3-oxoprop-1-enyl]-2,2-dimethylcyclopropane-1-carboxylate | 62.52 | 0.31 | XX |
| MOL002962 | (3S)-7-hydroxy-3-(2,3,4-trimethoxyphenyl)chroman-4-one | 48.23 | 0.33 | XX |
| MOL009849 | ZINC05223929 | 31.57 | 0.83 | XX |
| MOL001910 | 11alpha,12alpha-epoxy-3beta-23-dihydroxy-30-norolean-20-en-28,12beta-olide | 64.77 | 0.38 | BS |
| MOL001918 | paeoniflorgenone | 87.59 | 0.37 | BS |
| MOL001919 | (3S,5R,8R,9R,10S,14S)-3,17-dihydroxy-4,4,8,10,14-pentamethyl-2,3,5,6,7,9-hexahydro-1H-cyclopenta[a]phenanthrene-15,16-dione | 43.56 | 0.53 | BS |
| MOL001921 | Lactiflorin | 49.12 | 0.8 | BS |
| MOL001924 | paeoniflorin | 53.87 | 0.79 | BS |
| MOL001925 | paeoniflorin_qt | 68.18 | 0.4 | BS |
| MOL001928 | albiflorin_qt | 66.64 | 0.33 | BS |
| MOL001930 | benzoyl paeoniflorin | 31.27 | 0.75 | BS |
| MOL008006 | paryriogenin A | 41.41 | 0.76 | TC |
| MOL008020 | paryriogenin I | 45.26 | 0.79 | TC |
| MOL008025 | Tetrapanoside B_qt | 40.93 | 0.79 | TC |
| MOL012921 | stepharine | 31.55 | 0.33 | DZ |
| MOL012940 | Spiradine A | 113.52 | 0.61 | DZ |
| MOL012946 | zizyphus saponin I_qt | 32.69 | 0.62 | DZ |
| MOL012961 | jujuboside A_qt | 36.67 | 0.62 | DZ |
| MOL012976 | coumestrol | 32.49 | 0.34 | DZ |
| MOL012980 | Daechuine S6 | 46.48 | 0.79 | DZ |
| MOL012981 | Daechuine S7 | 44.82 | 0.83 | DZ |
| MOL012986 | Jujubasaponin V_qt | 36.99 | 0.63 | DZ |
| MOL012989 | Jujuboside C_qt | 40.26 | 0.62 | DZ |
| MOL012992 | Mauritine D | 89.13 | 0.45 | DZ |
| MOL001454 | berberine | 36.86 | 0.78 | DZ |
| MOL001522 | (S)-Coclaurine | 42.35 | 0.24 | DZ |
| MOL003410 | Ziziphin_qt | 66.95 | 0.62 | DZ |
| MOL004350 | Ruvoside_qt | 36.12 | 0.76 | DZ |
| MOL005360 | malkangunin | 57.71 | 0.63 | DZ |
| MOL000627 | Stepholidine | 33.11 | 0.54 | DZ |
| MOL007213 | Nuciferin | 34.43 | 0.4 | DZ |
| MOL000783 | Protoporphyrin | 30.86 | 0.56 | DZ |
| MOL000787 | Fumarine | 59.26 | 0.83 | DZ |
| MOL008034 | 21302-79-4 | 73.52 | 0.77 | DZ |
| MOL008647 | Moupinamide | 86.71 | 0.26 | DZ |
| MOL002773 | beta-carotene | 37.18 | 0.58 | DZ |
| MOL000096 | (-)-catechin | 49.68 | 0.24 | DZ |
| MOL013357 | (3S,6R,8S,9S,10R,13R,14S,17R)-17-[(1R,4R)-4-ethyl-1,5-dimethylhexyl]-10,13-dimethyl-2,3,6,7,8,9,11,12,14,15,16,17-dodecahydro-1H-cyclopenta[a]phenanthrene-3,6-diol | 34.37 | 0.78 | DZ |
| MOL004806 | euchrenone | 30.29 | 0.57 | GC |
| MOL004864 | 5,7-dihydroxy-3-(4-methoxyphenyl)-8-(3-methylbut-2-enyl)chromone | 30.49 | 0.41 | GC |
| MOL004985 | icos-5-enoic acid | 30.7 | 0.2 | GC |
| MOL004996 | gadelaidic acid | 30.7 | 0.2 | GC |
| MOL004805 | (2S)-2-[4-hydroxy-3-(3-methylbut-2-enyl)phenyl]-8,8-dimethyl-2,3-dihydropyrano[2,3-f]chromen-4-one | 31.79 | 0.72 | GC |
| MOL004814 | Isotrifoliol | 31.94 | 0.42 | GC |
| MOL004833 | Phaseolinisoflavan | 32.01 | 0.45 | GC |
| MOL004988 | Kanzonol F | 32.47 | 0.89 | GC |
| MOL001792 | DFV | 32.76 | 0.18 | GC |
| MOL004860 | licorice glycoside E | 32.89 | 0.27 | GC |
| MOL004882 | Licocoumarone | 33.21 | 0.36 | GC |
| MOL004905 | 3,22-Dihydroxy-11-oxo-delta(12)-oleanene-27-alpha-methoxycarbonyl-29-oic acid | 34.32 | 0.55 | GC |
| MOL004935 | Sigmoidin-B | 34.88 | 0.41 | GC |
| MOL004978 | 2-[(3R)-8,8-dimethyl-3,4-dihydro-2H-pyrano[6,5-f]chromen-3-yl]-5-methoxyphenol | 36.21 | 0.52 | GC |
| MOL004945 | (2S)-7-hydroxy-2-(4-hydroxyphenyl)-8-(3-methylbut-2-enyl)chroman-4-one | 36.57 | 0.32 | GC |
| MOL004917 | glycyroside | 37.25 | 0.79 | GC |
| MOL004957 | HMO | 38.37 | 0.21 | GC |
| MOL004991 | 7-Acetoxy-2-methylisoflavone | 38.92 | 0.26 | GC |
| MOL004884 | Licoisoflavone B | 38.93 | 0.55 | GC |
| MOL004989 | 6-prenylated eriodictyol | 39.22 | 0.41 | GC |
| MOL004815 | (E)-1-(2,4-dihydroxyphenyl)-3-(2,2-dimethylchromen-6-yl)prop-2-en-1-one | 39.62 | 0.35 | GC |
| MOL004980 | Inflacoumarin A | 39.71 | 0.33 | GC |
| MOL000497 | licochalcone a | 40.79 | 0.29 | GC |
| MOL004924 | (-)-Medicocarpin | 40.99 | 0.95 | GC |
| MOL005013 | 18α-hydroxyglycyrrhetic acid | 41.16 | 0.71 | GC |
| MOL005008 | Glycyrrhiza flavonol A | 41.28 | 0.6 | GC |
| MOL004883 | Licoisoflavone | 41.61 | 0.42 | GC |
| MOL003896 | 7-Methoxy-2-methyl isoflavone | 42.56 | 0.2 | GC |
| MOL004915 | Eurycarpin A | 43.28 | 0.37 | GC |
| MOL004966 | 3'-Hydroxy-4'-O-Methylglabridin | 43.71 | 0.57 | GC |
| MOL004866 | 2-(3,4-dihydroxyphenyl)-5,7-dihydroxy-6-(3-methylbut-2-enyl)chromone | 44.15 | 0.41 | GC |
| MOL004948 | Isoglycyrol | 44.7 | 0.84 | GC |
| MOL004828 | Glepidotin A | 44.72 | 0.35 | GC |
| MOL004949 | Isolicoflavonol | 45.17 | 0.42 | GC |
| MOL004811 | Glyasperin C | 45.56 | 0.4 | GC |
| MOL004974 | 3'-Methoxyglabridin | 46.16 | 0.57 | GC |
| MOL004911 | Glabrene | 46.27 | 0.44 | GC |
| MOL004898 | (E)-3-[3,4-dihydroxy-5-(3-methylbut-2-enyl)phenyl]-1-(2,4-dihydroxyphenyl)prop-2-en-1-one | 46.27 | 0.31 | GC |
| MOL004961 | Quercetin der. | 46.45 | 0.33 | GC |
| MOL000417 | Calycosin | 47.75 | 0.24 | GC |
| MOL004913 | 1,3-dihydroxy-9-methoxy-6-benzofurano[3,2-c]chromenone | 48.14 | 0.43 | GC |
| MOL004827 | Semilicoisoflavone B | 48.78 | 0.55 | GC |
| MOL004857 | Gancaonin B | 48.79 | 0.45 | GC |
| MOL002565 | Medicarpin | 49.22 | 0.34 | GC |
| MOL004848 | licochalcone G | 49.25 | 0.32 | GC |
| MOL000354 | isorhamnetin | 49.6 | 0.31 | GC |
| MOL005016 | Odoratin | 49.95 | 0.3 | GC |
| MOL005001 | Gancaonin H | 50.1 | 0.78 | GC |
| MOL004820 | kanzonols W | 50.48 | 0.52 | GC |
| MOL000239 | Jaranol | 50.83 | 0.29 | GC |
| MOL004856 | Gancaonin A | 51.08 | 0.4 | GC |
| MOL003656 | Lupiwighteone | 51.64 | 0.37 | GC |
| MOL004885 | licoisoflavanone | 52.47 | 0.54 | GC |
| MOL004912 | Glabrone | 52.51 | 0.5 | GC |
| MOL004879 | Glycyrin | 52.61 | 0.47 | GC |
| MOL004910 | Glabranin | 52.9 | 0.31 | GC |
| MOL004908 | Glabridin | 53.25 | 0.47 | GC |
| MOL004993 | 8-prenylated eriodictyol | 53.79 | 0.4 | GC |
| MOL005020 | dehydroglyasperins C | 53.82 | 0.37 | GC |
| MOL005018 | Xambioona | 54.85 | 0.87 | GC |
| MOL005012 | Licoagroisoflavone | 57.28 | 0.49 | GC |
| MOL004838 | 8-(6-hydroxy-2-benzofuranyl)-2,2-dimethyl-5-chromenol | 58.44 | 0.38 | GC |
| MOL005003 | Licoagrocarpin | 58.81 | 0.58 | GC |
| MOL004328 | naringenin | 59.29 | 0.21 | GC |
| MOL004849 | 3-(2,4-dihydroxyphenyl)-8-(1,1-dimethylprop-2-enyl)-7-hydroxy-5-methoxy-coumarin | 59.62 | 0.43 | GC |
| MOL004824 | (2S)-6-(2,4-dihydroxyphenyl)-2-(2-hydroxypropan-2-yl)-4-methoxy-2,3-dihydrofuro[3,2-g]chromen-7-one | 60.25 | 0.63 | GC |
| MOL005000 | Gancaonin G | 60.44 | 0.39 | GC |
| MOL004907 | Glyzaglabrin | 61.07 | 0.35 | GC |
| MOL004835 | Glypallichalcone | 61.6 | 0.19 | GC |
| MOL004914 | 1,3-dihydroxy-8,9-dimethoxy-6-benzofurano[3,2-c]chromenone | 62.9 | 0.53 | GC |
| MOL004855 | Licoricone | 63.58 | 0.47 | GC |
| MOL004829 | Glepidotin B | 64.46 | 0.34 | GC |
| MOL004808 | glyasperin B | 65.22 | 0.44 | GC |
| MOL004903 | liquiritin | 65.69 | 0.74 | GC |
| MOL004863 | 3-(3,4-dihydroxyphenyl)-5,7-dihydroxy-8-(3-methylbut-2-enyl)chromone | 66.37 | 0.41 | GC |
| MOL000392 | formononetin | 69.67 | 0.21 | GC |
| MOL004959 | 1-Methoxyphaseollidin | 69.98 | 0.64 | GC |
| MOL004941 | (2R)-7-hydroxy-2-(4-hydroxyphenyl)chroman-4-one | 71.12 | 0.18 | GC |
| MOL005007 | Glyasperins M | 72.67 | 0.59 | GC |
| MOL000500 | Vestitol | 74.66 | 0.21 | GC |
| MOL001484 | Inermine | 75.18 | 0.54 | GC |
| MOL004810 | glyasperin F | 75.84 | 0.54 | GC |
| MOL004841 | Licochalcone B | 76.76 | 0.19 | GC |
| MOL005017 | Phaseol | 78.77 | 0.58 | GC |
| MOL004891 | shinpterocarpin | 80.3 | 0.73 | GC |
| MOL004904 | licopyranocoumarin | 80.36 | 0.65 | GC |
| MOL004990 | 7,2',4'-trihydroxy－5-methoxy-3－arylcoumarin | 83.71 | 0.27 | GC |
| MOL002311 | Glycyrol | 90.78 | 0.67 | GC |

DL = drug-likeness, OB = oral bioavailability.
